# Supplementary material for: Loss of ANCO1 Expression Regulates Chromatin Accessibility and Drives Progression of Early-Stage Triple-Negative Breast Cancer
Source: Int J Mol Sci. 2023 Jul 15;24(14):11505. doi: 10.3390/ijms241411505 (PMC10380654; doi:10.3390/ijms241411505)
Supplement: Supplementary file 1 [file ijms-24-11505-s001.zip › Captions for supplementary materials.pdf]

Supplementary Figure S1.
(**A**) ANCO1 mRNA levels in the different IHC subtypes of breast cancer. TCGA and Metabric datasets were used for this analysis. n= 526 and 1,257, respectively. ANOVA test was used for statistical analysis. (**B**) Representative images of ANCO1 IHC staining in mouse xenograft tumors. Scale bars, 200 μm. (**C**) Recurrence-free survival probability based on nuclear ANCO1 protein levels in all subtypes of breast cancer in the TMA. ANCO1 level was stratified by IQR. n = 146 patients. (**D**) Recurrence-free survival probability based on cytoplasmic ANCO1 protein levels in all subtypes of breast cancer in the TMA. ANCO1 level was stratified by IQR. n = 146 patients. (**E**) Overall survival of patients with TNBC based on cytoplasmic ANCO1 protein expression. ANCO1 level was stratified by IQR. n = 48 patients.

Supplementary Figure S2.
(**A**) Representative images of ANCO1 and DAPI IF staining in DCIS control and shANCO1 cells. Scale bars, 50 μm. (**B**) Bright-field images showing changes in size and shape of shANCO1 10A and DCIS cell lines. Scale bars as indicated. Areas in inset boxes are enlarged in Fig. 2D. (**C**) Apoptotic analysis of control and shANCO1 10A and DCIS cells by Annexin staining and flow cytometry. Three biological replicates per group were quantified. Unpaired t-tests were used for statistical analysis in panel C. *, $P < 0.05$; **, $P < 0.01$; ***, $P < 0.001$; ****, $P < 0.0001$; ns, $P > 0.05$.

Supplementary Figure S3.
(**A**) A schematic illustrating 3D culture system of spheres in Matrigel. Culture slide chambers were first coated with 100% Matrigel. Cells were suspended in 2% Matrigel and plated on top of pure Matrigel layer to form spheres. (**B**) A schematic illustrating 3D culture system of spheres that were aggregated in U-shaped wells and then embedded in a Matrigel and Collagen I mixture.

Supplementary Figure S4.
(**A**) A schematic illustrating peak calling and definition of ANCO1-regulated H3K27Ac peaks. Differential binding was identified by comparing H3K27Ac chromatin engagement between shANCO1 and control groups. Enriched binding was confirmed by comparing immunoprecipitated samples with input controls. The identified up- and down-regulated H3K27Ac peaks in shANCO1 groups were defined as ANCO1-regulated peaks.

Supplementary Figure S5.
(**A**) Heatmaps showing top-ranked differentially expressed genes in 10A and DCIS shANCO1 versus control cell lines in 2D culture. Red and blue colors indicate high and low expressions respectively ($P < 0.05$). (**B**) Volcano plots showing differentially expressed genes in 10A and DCIS shANCO1 versus control cell lines, in 2D cell culture. Red and blue colors indicate up- or down-regulated genes respectively. Three shANCO1 lines were combined for this analysis. Three biological replicates per condition. (**C**) Selected GSEA results of signaling pathways enriched in ANCO1 knockdown 10A and DCIS cell lines in 2D culture. (**D**) Heatmaps showing top-ranked differentially expressed genes in 10A and DCIS shANCO1 versus control cell lines in 3D culture ($P < 0.05$). (**E**) Volcano plots showing differentially expressed genes in 10A and DCIS shANCO1 versus control 3D spheres. Two shANCO1 lines were combined for this analysis. (**F**) GSEA of mTORC1 signaling in shANCO1 10A and DCIS spheres compared to their respective controls.

Supplementary Table S1
The demographics of patients in the TMA cohort. Total n = 150 patients.

Supplementary Table S2
Counts of polyploid cells in shANCO1 versus control DCIS cells from FISH analysis.

Supplementary Table S3
Lists of enriched signaling pathways discovered in genes annotated to ANCO1-regulated H3K27Ac peaks in 10A and DCIS cell lines. Analysis was performed in DAVID.

Supplementary Table S4
Lists of enriched motifs discovered in up-regulated H3K27Ac peaks in shANCO1 versus control 10A and DCIS cells. Analysis was performed using the Known Motif method in HOMER.

Supplementary Table S5
Lists of differentially expressed genes in shANCO1 10A and DCIS cell lines compared to their respective controls in 2D and 3D cultures, with the absolute value of log2FC greater than 1.2.

Supplementary Table S6
Lists of regulated signaling pathways in shANCO1 versus control 10A and DCIS cells, in 2D and 3D cultures. Analysis was performed in GSEA.

Supplementary Table S7
Lists of enriched signaling pathways in upregulated genes (Log2FC > 1.2) with increased H3K27Ac engagement in shANCO1 10A and DCIS cells compared to respective controls. Analysis was performed in IPA.
