# Supplementary material for: Loss of ANCO1 Expression Regulates Chromatin Accessibility and Drives Progression of Early-Stage Triple-Negative Breast Cancer
Source: Int J Mol Sci. 2023 Jul 15;24(14):11505. doi: 10.3390/ijms241411505 (PMC10380654; doi:10.3390/ijms241411505)
Supplement: Supplementary file 1 [file ijms-24-11505-s001.zip › Figure_S1.pdf]

Figure S1

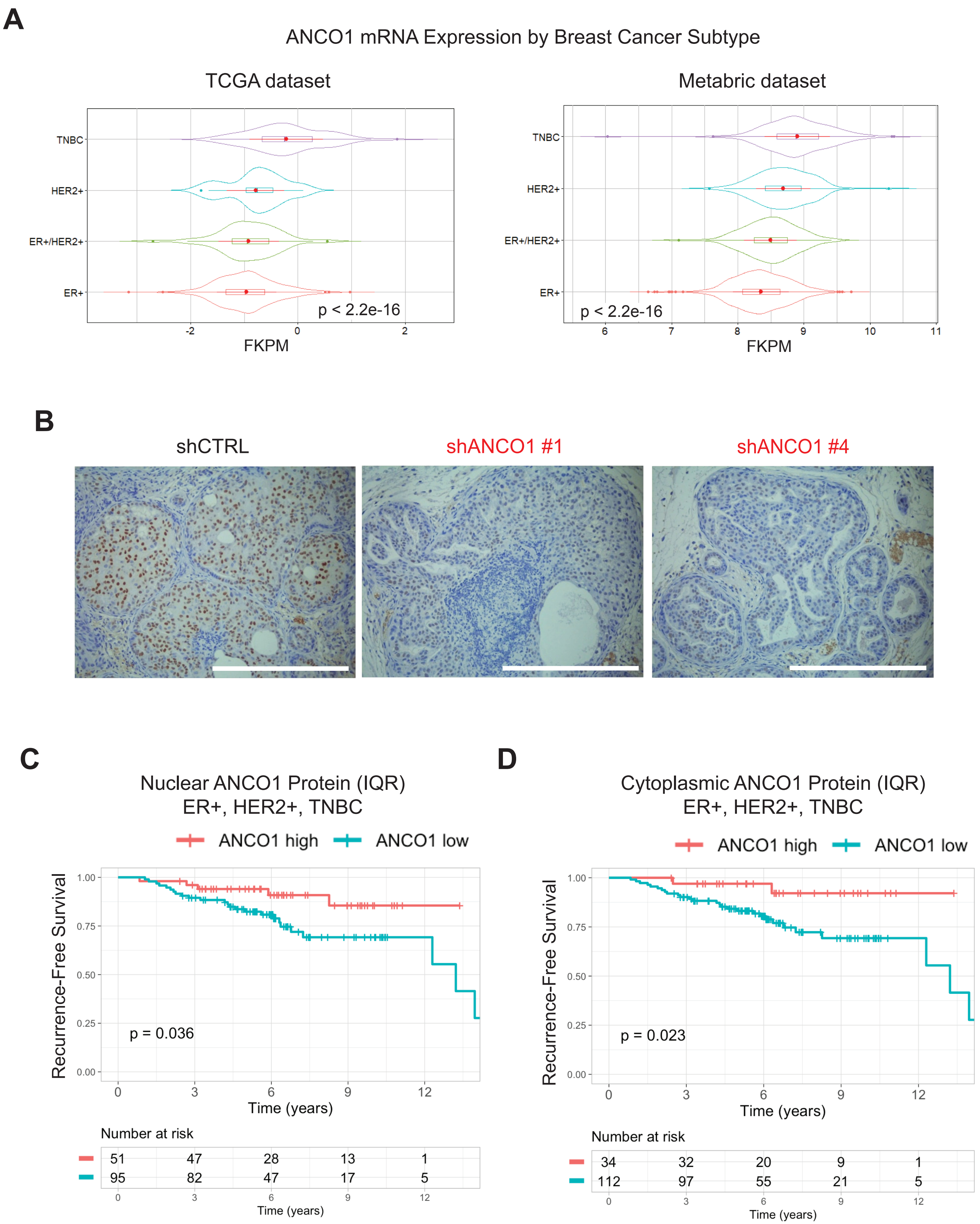

A
ANCO1 mRNA Expression by Breast Cancer Subtype
TCGA dataset
Metabric dataset
TNBC
HER2+
ER+/HER2+
ER+
p < 2.2e-16
FKPM
-2
0
2
6
7
8
9
10
11
B
shCTRL
shANCO1 #1
shANCO1 #4
C
Nuclear ANCO1 Protein (IQR)
ER+, HER2+, TNBC
ANCO1 high
ANCO1 low
Recurrence-Free Survival
1.00
0.75
0.50
0.25
0.00
p = 0.036
0
3
6
9
12
Time (years)
Number at risk
51 47 28 13 1
95 82 47 17 5
D
Cytoplasmic ANCO1 Protein (IQR)
ER+, HER2+, TNBC
ANCO1 high
ANCO1 low
Recurrence-Free Survival
p = 0.023
Time (years)
Number at risk
34 32 20 9 1
112 97 55 21 5

E

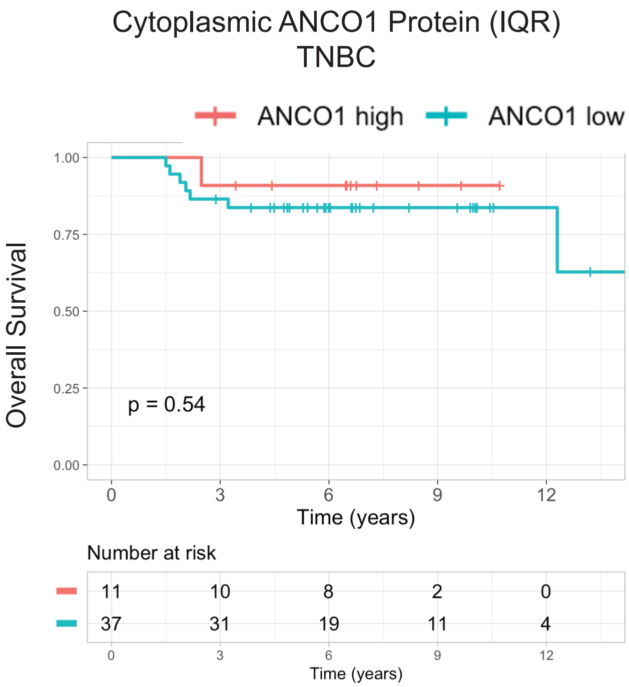

Figure s1: (A) ANCO1 mRNA levels in the different IHC subtypes of breast cancer. TCGA and Metabric datasets were used for this analysis. n= 526 and 1,257, respectively. ANOVA test was used for statistical analysis. (B) Representative images of ANCO1 IHC staining in mouse xenograft tumors. Scale bars, 200 μm. (C) Recurrence-free survival probability based on nuclear ANCO1 protein levels in all subtypes of breast cancer in the TMA. ANCO1 level was stratified by IQR. n = 146 patients. (D) Recurrence-free survival probability based on cytoplasmic ANCO1 protein levels in all subtypes of breast cancer in the TMA. ANCO1 level was stratified by IQR. n = 146 patients. (E) Overall survival of patients with TNBC based on cytoplasmic ANCO1 protein expression. ANCO1 level was stratified by IQR. n = 48 patients.
