# Supplementary material for: Loss of ANCO1 Expression Regulates Chromatin Accessibility and Drives Progression of Early-Stage Triple-Negative Breast Cancer
Source: Int J Mol Sci. 2023 Jul 15;24(14):11505. doi: 10.3390/ijms241411505 (PMC10380654; doi:10.3390/ijms241411505)
Supplement: Supplementary file 1 [file ijms-24-11505-s001.zip › Figure_S2.pdf]

Figure S2

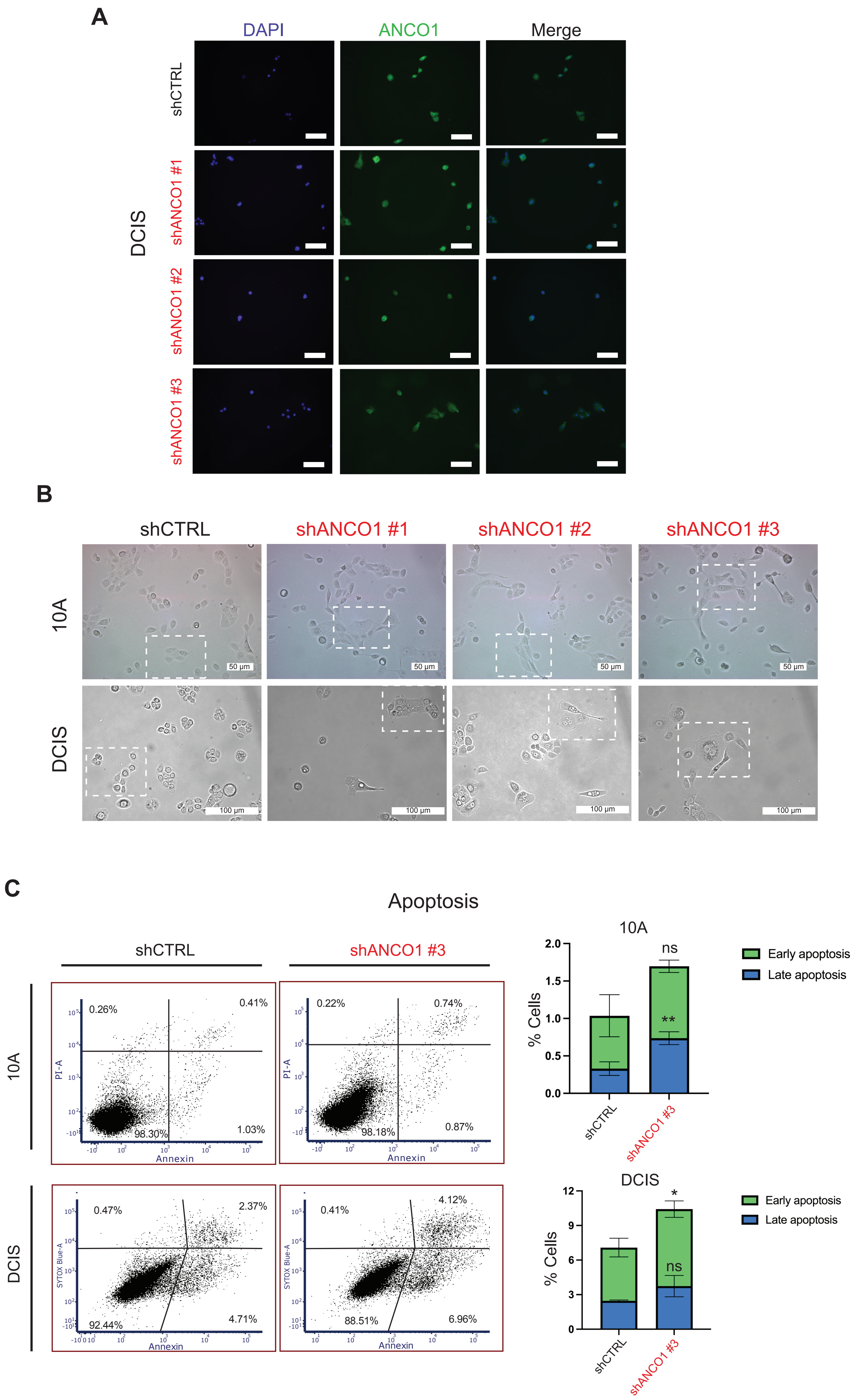

Figure s2:(A) Representative images of ANCO1 and DAPI IF staining in DCIS control and shANCO1 cells. Scale bars, 50 µ m. (B) Bright-field images showing changes in size and shape of shANCO1 10A and DCIS cell lines. Scale bars as indicated. Areas in inset boxes are enlarged in Fig. 2D. (C) Apoptotic analysis of control and shANCO1 10A and DCIS cells by Annexin staining and flow cytometry. Three biological replicates per group were quantified. Unpaired t-tests were used for statistical analysis in panel C. *, $P < 0.05$; **, $P < 0.01$; ***, $P < 0.001$; ****, $P < 0.0001$; ns, $P > 0.05$.
