# Supplementary material for: Loss of ANCO1 Expression Regulates Chromatin Accessibility and Drives Progression of Early-Stage Triple-Negative Breast Cancer
Source: Int J Mol Sci. 2023 Jul 15;24(14):11505. doi: 10.3390/ijms241411505 (PMC10380654; doi:10.3390/ijms241411505)
Supplement: Supplementary file 1 [file ijms-24-11505-s001.zip › Figure_S3.pdf]

Figure S3

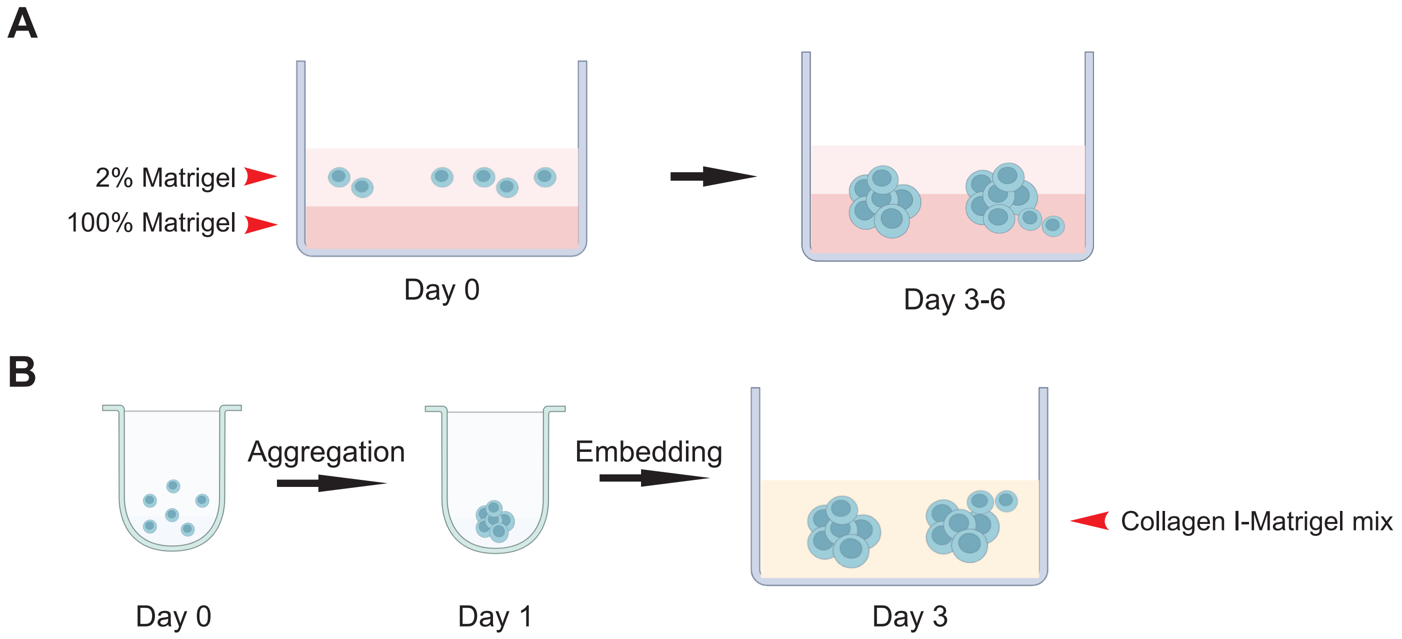

Figure s3:(A) A schematic illustrating 3D culture system of spheres in Matrigel. Culture slide chambers were first coated with 100% Matrigel. Cells were suspended in 2% Matrigel and plated on top of pure Matrigel layer to form spheres. (B) A schematic illustrating 3D culture system of spheres that were aggregated in U-shaped wells and then embedded in a Matrigel and Collagen I mixture.
