# Supplementary material for: Loss of ANCO1 Expression Regulates Chromatin Accessibility and Drives Progression of Early-Stage Triple-Negative Breast Cancer
Source: Int J Mol Sci. 2023 Jul 15;24(14):11505. doi: 10.3390/ijms241411505 (PMC10380654; doi:10.3390/ijms241411505)
Supplement: Supplementary file 1 [file ijms-24-11505-s001.zip › Figure_S4.pdf]

Figure S4

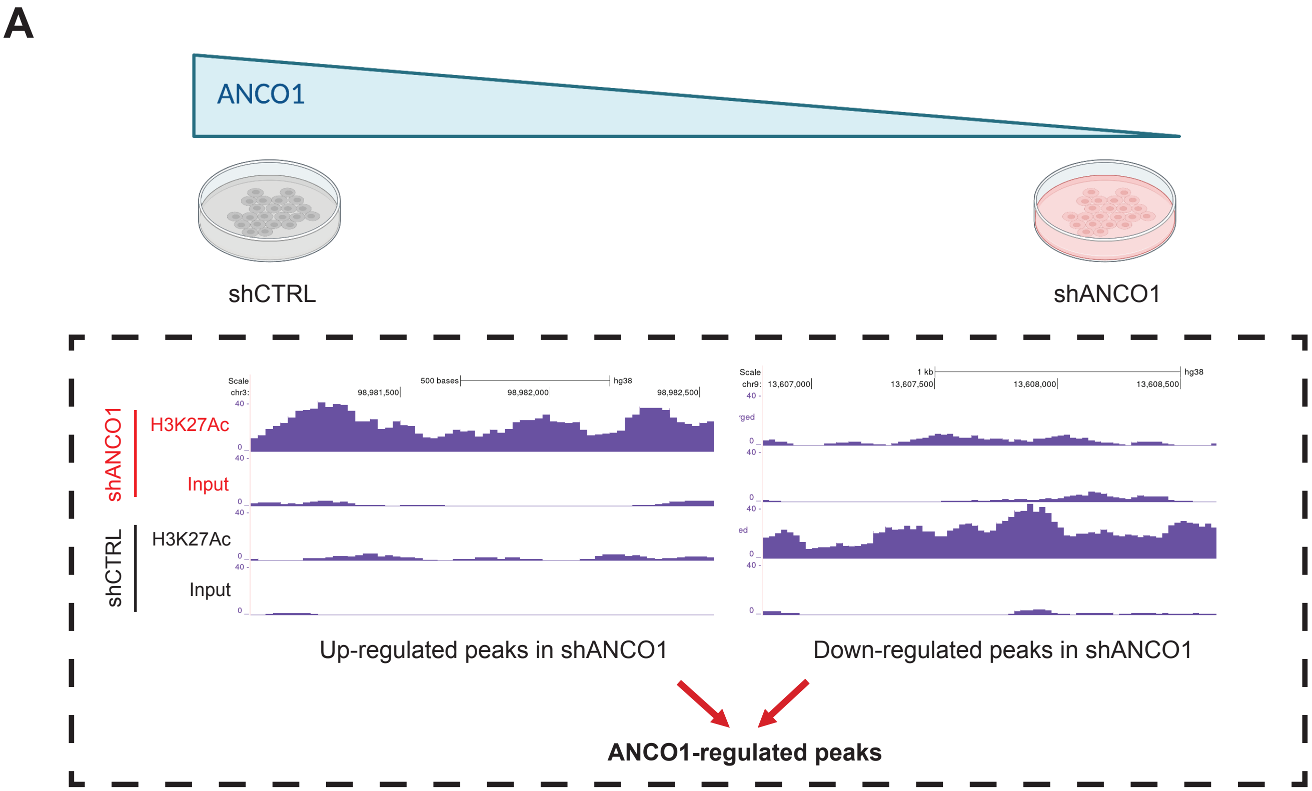

Figure s4: (A) A schematic illustrating peak calling and definition of ANCO1-regulated H3K27Ac peaks. Differential binding was identified by comparing H3K27Ac chromatin engagement between shANCO1 and control groups. Enriched binding was confirmed by comparing immunoprecipitated samples with input controls. The identified up- and down-regulated H3K27Ac peaks in shANCO1 groups were defined as ANCO1-regulated peaks.
