# Supplementary material for: Loss of ANCO1 Expression Regulates Chromatin Accessibility and Drives Progression of Early-Stage Triple-Negative Breast Cancer
Source: Int J Mol Sci. 2023 Jul 15;24(14):11505. doi: 10.3390/ijms241411505 (PMC10380654; doi:10.3390/ijms241411505)
Supplement: Supplementary file 1 [file ijms-24-11505-s001.zip › Figure_S5.pdf]

Figure S5

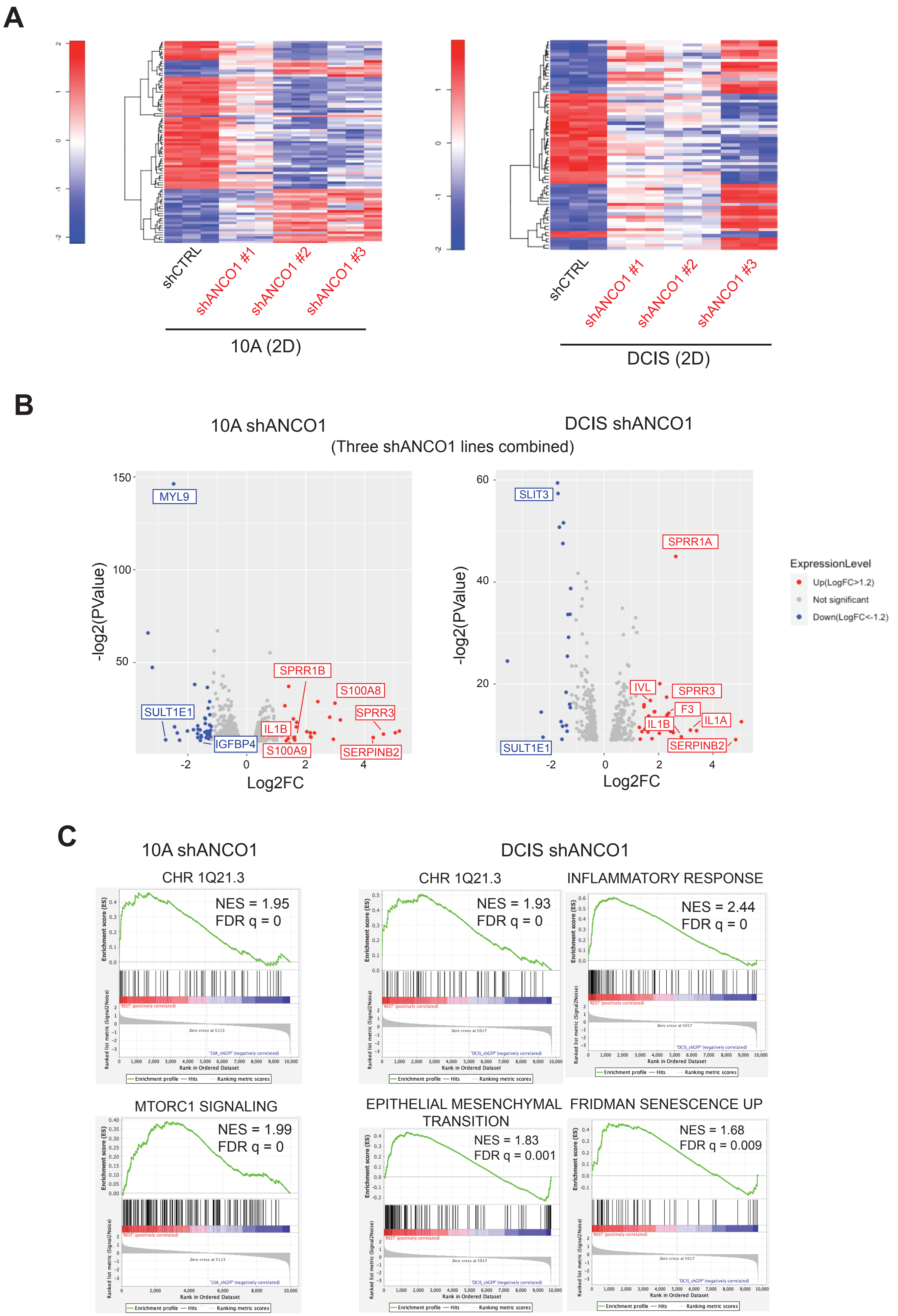
A
shCTRL
shANCO1 #1
shANCO1 #2
shANCO1 #3
10A (2D)
DCIS (2D)
B
10A shANCO1
DCIS shANCO1
(Three shANCO1 lines combined)
-log2(PValue)
Log2FC
MYL9
SULT1E1
IGFBP4
SPRR1B
S100A8
SPRR3
IL1B
S100A9
SERPINB2
SLIT3
SPRR1A
IVL
F3
IL1A
ExpressionLevel
Up(LogFC>1.2)
Not significant
Down(LogFC<-1.2)
C
10A shANCO1
DCIS shANCO1
CHR 1Q21.3
NES = 1.95
FDR q = 0
CHR 1Q21.3
NES = 1.93
FDR q = 0
INFLAMMATORY RESPONSE
NES = 2.44
FDR q = 0
MTORC1 SIGNALING
NES = 1.99
FDR q = 0
EPITHELIAL MESENCHYMAL TRANSITION
NES = 1.83
FDR q = 0.001
FRIDMAN SENESCENCE UP
NES = 1.68
FDR q = 0.009

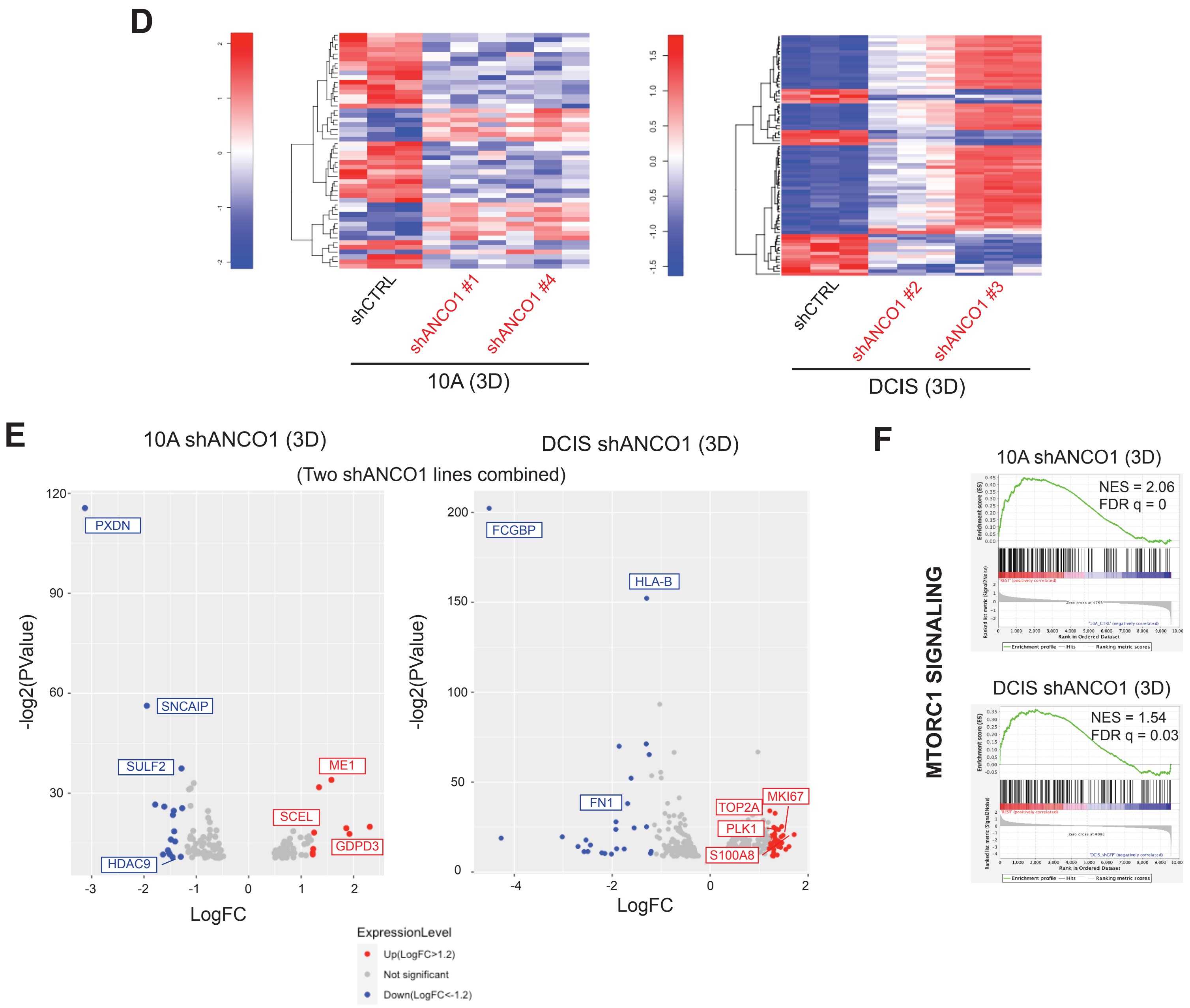

Figure s5:(A) Heatmaps showing top-ranked differentially expressed genes in 10A and DCIS shANCO1 versus control cell lines in 2D culture. Red and blue colors indicate high and low expressions respectively (P < 0.05). (B) Volcano plots showing differentially expressed genes in 10A and DCIS shANCO1 versus control cell lines, in 2D cell culture. Red and blue colors indicate up- or down-regulated genes respectively. Three shANCO1 lines were combined for this analysis. Three biological replicates per condition. (C) Selected GSEA results of signaling pathways enriched in ANCO1 knockdown 10A and DCIS cell lines in 2D culture. (D) Heatmaps showing top-ranked differentially expressed genes in 10A and DCIS shANCO1 versus control cell lines in 3D culture (P < 0.05). (E) Volcano plots showing differentially expressed genes in 10A and DCIS shANCO1 versus control 3D spheres. Two shANCO1 lines were combined for this analysis. (F) GSEA of mTORC1 signaling in shANCO1 10A and DCIS spheres compared to their respective controls.
