# Supplementary figures and images for: Loss of ANCO1 Expression Regulates Chromatin Accessibility and Drives Progression of Early-Stage Triple-Negative Breast Cancer
Source: Int J Mol Sci. 2023 Jul 15;24(14):11505. doi: 10.3390/ijms241411505 (PMC10380654; doi:10.3390/ijms241411505)

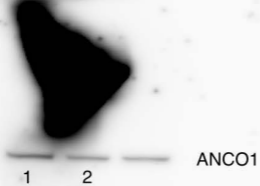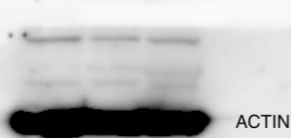

1: 10A\_shCTRL  
2: 10A\_shANCO1 #4

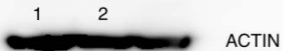

1: 10A\_shCTRL  
2: 10A\_shANCO1 #4

Supplement: Supplementary file 1 [file ijms-24-11505-s001.zip › Original_western blot_10A_shANCO1 #4.pdf]

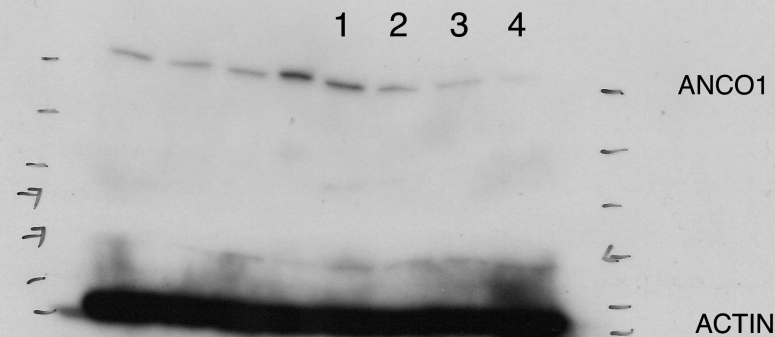

1. 10A\_shCTRL
2. 10A\_shANCO1 #1
3. 10A\_shANCO1 #2
4. 10A\_shANCO1 #3

[min 100]

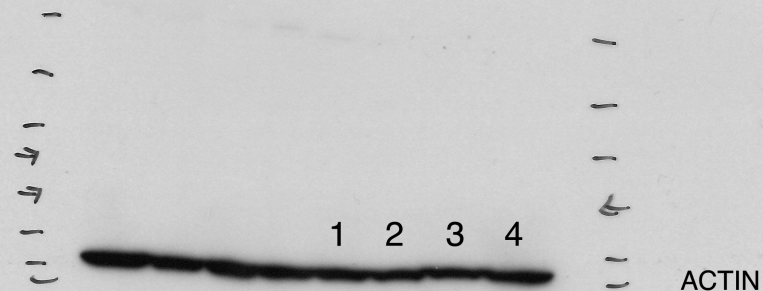

1. 10A\_shCTRL
2. 10A\_shANCO1 #1
3. 10A\_shANCO1 #2
4. 10A\_shANCO1 #3

35 (2)

Supplement: Supplementary file 1 [file ijms-24-11505-s001.zip › Original_western blot_10A_shANCO1_#1_2_3_1min_3s_exposure.pdf]
